# Patients’ views of the healthcare services during power outages in Pretoria Primary Healthcare

**DOI:** 10.4102/jcmsa.v4i1.254

**Published:** 2026-01-21

**Authors:** Thando T. Mnisi, Tombo Bongongo, Adeline M. Nkoane

**Affiliations:** 1 Department of Family Medicine and Primary Health Care, School of Medicine, Sefako Makgatho Health Sciences University, Pretoria, South Africa

**Keywords:** patients, healthcare services, power outages, primary healthcare, Pretoria

## Abstract

**Background:**

Power outages are prevalent in low- and middle-income countries. They cause a partial or complete interruption of power supply for electrical customers, including the public, businesses and essential services. This study explored the experiences of patients at Pretoria Primary Healthcare facilities during power outages.

**Methods:**

This was a qualitative study with a descriptive phenomenology design using focus group discussions in three Community Health Centres (Soshanguve 3, Phedisong 4 and Kgabo) of Pretoria.

**Results:**

Of the 19 respondents, 12 (63.12%) were women and 7 (36.84%) were men. The average age was 50.52, with a minimum of 30 years old and a maximum of 74 years old. Four themes emerged from the analysis: restricted healthcare services during power outages, alternatives available during power outages, patient perceptions of staff behaviour and attitudes during power outages and recommendations for mitigating the effects of power outages. These were expanded upon two sub-themes: previous attempts to involve staff for improvement and strategies for minimising the consequences of power outages in clinics.

**Conclusion:**

In the selected Pretoria Primary Healthcare facilities, power outages disrupted medical services such as diagnostic, treatment and administration, resulting in extended waiting times and poor health outcomes. Generators and other restricted alternatives for resolving the problem were used.

**Contribution:**

Negative health effects can be prevented if the current issue is considered in the healthcare system.

## Introduction

A power outage is defined as a partial or total loss of power supply to an electrical consumer, such as a population, businesses or important services. Power outages are a worldwide occurrence.^1^

A cable cut in the Berlin district in 2019 led to a multi-day power outage affecting 30 000 households and 70 000 individuals. This outage caused significant difficulties, necessitating the reevaluation of medical needs within an operational management system. Particular attention was given to at-risk patients, including those requiring home ventilation or using artificial hearts. The power disruption also required the relocation of residents from nursing homes and impacted an intensive or intermediate care unit with 23 patients.^2^ Few years later, a cohort study following Hurricane Irma in a Florida nursing home, in the United States (US), investigated the link between power outages and increased death and morbidity rates among residents. The study involved 27 892 residents exposed to power outages and 26 203 residents who were not. The populations were comparable in terms of ownership, size and quality rating. Findings indicated that residents experiencing power outages had higher rates of illness and mortality compared to those who did not. Most affected residents were between 65 and 74 years old, with a particular focus on excessive ambient heat as a potential causal factor.^3^

A study in Malawi investigated electricity access in 44 healthcare facilities, finding that under a third possess a backup power source and experience weekly outages averaging 10 h. These power disruptions negatively affect provider safety, lighting, work environments, water systems and sanitation. In this country, power outages significantly hamper healthcare provision because of unreliable grids and frequent blackouts. While some facilities received advance notice of scheduled outages, enabling better preparedness, many did not negatively affect both healthcare providers and patients. The study recommends tailoring energy access solutions to the specific needs of various facility types to enhance care quality.^4^ Concurrently in Kenya, a 2020 study assessed therapeutic oxygen availability and electrical supply reliability in rural healthcare settings. Among 57 patients with hypoxaemia (children and youth), power outages exceeding 17 min, occurring more than 6 days, correlated with a 19% case fatality rate. Additionally, 32% of patients experienced oxygen interruptions of at least 11 min, and 28% relied on backup cylinders. The research concluded that high hypoxaemia-related mortality in resource-limited healthcare institutions may be attributable to oxygen insecurity, stemming from inadequate equipment and/or unreliable electricity.^5^

A 2014–2015 study in Cape Town, South Africa, revealed that power outages led to a 10% rise in hospital admissions from June to May, suggesting a significant, though largely unquantified, impact on children’s health. The study documented an average treatment effect of 6.50 admissions daily because of power interruptions, with weekdays experiencing a 12% increase and specific diagnoses a 14% increase, thereby supporting the notion of heightened health risks for children.^6^ Further evidence from another South African publication indicated serious repercussions of power failures and inadequate backup systems in hospitals. This was supported by US data showing a 28% increase in mortality during mass power outages in 2003 and a 43% daily increase in hospital mortality when outages surpassed 2 h.^7^ Power outages significantly impact South Africa’s healthcare system, particularly smaller facilities, with a 2-h outage potentially causing a 43% increase in mortality. The public healthcare system, serving over 80% of the population through approximately 3000 state-run clinics and 420 state-run hospitals, is vulnerable. While tertiary and secondary level hospitals often possess generator banks, smaller facilities are frequently neglected in preparedness strategies.^7^

Given the impact that power outages may have on society, particularly the healthcare sector, the current study explored patients’ views on healthcare services during power outages in Pretoria primary care.

## Research methods and design

### Study design

This was a qualitative study using a descriptive phenomenology design, with three focus group discussions (FGDs) of six people per Community Health Centre (CHC).

### Study setting

Three CHCs in region 1 of the Tshwane Health District, in Pretoria, served as the study’s sites. This comprises Kgabo, Phedisong 4, and Soshanguve 3 CHC. Located in the northwest of the Gauteng province, these CHCs provide services to an estimated 997 000 people, mostly Africans who speak Tswana and English. These healthcare facilities were selected because they have family physicians, and they were part of the researcher’s rotation.

### Study population, sampling technique and sample size

Soshanguve 3, Phedisong 4 and Kgabo are the three CHCs from which participants were gathered. A focus group with a minimum of 5 to 10 participants was held at each CHC. A retired female nurse with expertise in gathering qualitative data was recruited by the researcher to serve as a research assistant (RA). Both Tswana and English, the languages used in the research area, are spoken fluently by the RA, and they were tasked to outline the aim and the objectives of the study to the prospective participants before conducting interviews.

Patients were informed about the study in the waiting areas of each CHC. After obtaining all necessary medical care, the first 10 patients were asked to join the RA, and interested volunteers were selected. At each CHC, before data collection, participants were informed about the study, their concerns were addressed, and they were given informed consent forms to sign before interviews.

All patients aged 18 years and above who had been receiving care at the CHC for at least 2 years before the data collection date and who were willing to participate in the study were eligible, while adult patients who needed urgent medical attention were not selected. Therefore, with this purposive sampling, the sample size was 19 participants (*n* = 19).

### Data collection

In the designated room provided by the facility management of each CHC, the researcher introduced the RA, the study, its objectives and its aim. The researcher explained that it was a focus group discussion in which anyone who wanted to speak had to ask permission from the RA, the time was 1 h, participation was not remunerated, if someone felt uncomfortable, he or she could excuse himself or herself, no identity or documents would be used, and each participant would be given a letter and a number such as P1, P2, P3, P4, P5, etc. In the focus group discussion, P represents the participant, and 1, 2 and 3 denote the number. The researcher left the room, and the RA will be introducing the problem or question, and whoever has an answer can request to speak. The interviews were conducted in English and Tswana as these are the two spoken languages in the study area. They were audio-taped using the researcher’s phone.

### Data analysis

The socio-demographic factors of the participants were captured and exported to STATA-18 for descriptive analysis and presented graphically. The qualitative raw data from the three focus groups were converted from audio to text using Microsoft Word and subsequently checked for accuracy through the editing and cleaning process. The cleaned transcripts were exported to NVivo 14 for thematic analysis, whereby recurring codes were formed and merged to develop themes and sub-themes. Initial reading of the transcripts allowed for the initial development and definition of codes. Subsequent transcripts were used to merge existing codes, and where necessary, new codes were developed. The final codes were grouped accordingly to form themes and sub-themes related to the study objectives, aims and questions. The final number of five themes and five sub-themes emerged from the analysis.

### Ethical considerations

The Tshwane Research Committee (TRC) granted permission to the researcher, and SMUREC (Sefako Makgatho Health Sciences University Research and Ethics Committee: SMUREC/M/284/2024:PG) issued a clearance certificate. Anonymity and confidentiality were maintained throughout the entire study process.

## Results

### Socio-demographics

Of the 19 participants, the mean age was 50.52 with an s.d. of 11.85, ranging from 30 to 74. Six participants were from Soshanguve 3 CHC, six from Phedisong 4 CHC and seven from Kgabo CHC. Table 1 that follows shows the participants’ details:

**TABLE 1:** Participant details.

Participants per clinic	Age (years)	Gender
**Kgabo CHC**		
Participant 1	41	Female
Participant 2	52	Male
Participant 3	42	Female
Participant 4	39	Female
Participant 5	43	Female
Participant 6	32	Male
Participant 7	54	Female
**Phedisong 4 CHC**		
Participant 1	53	Female
Participant 2	60	Male
Participant 3	60	Male
Participant 4	39	Male
Participant 5	57	Female
Participant 6	62	Female
**Soshanguve 3 CHC**		
Participant 1	54	Male
Participant 2	30	Female
Participant 3	74	Female
Participant 4	69	Female
Participant 5	50	Female
Participant 6	49	Male

CHC, community health centre.

While the percentages of the samples from Phedisong 4 CHC and Soshanguve 3 CHC were comparable (*n* = 6; 31.6%), Kgabo CHC had the highest proportion (*n* = 7; 36.8%) with seven individuals. Most participants – 63.1% of the sample – were female, as shown in Table 2.

**TABLE 2:** Participants per CHC and gender details.

Socio-demographics per clinic	Frequency	%
**Clinic name**		
Kgabo CHC	7	36.84
Phedisong 4 CHC	6	31.58
Soshanguve 3 CHC	6	31.58
**Gender**		
Female	12	63.12
Male	7	36.84

CHC, community health centre.

## Findings from focus group discussions

### Presentation of themes and sub-themes

Nineteen people participated in all, split up across three focus groups from three distinct medical facilities. Thematic analysis was used to create codes, sub-themes and overarching themes from each focus group discussion by classifying the participant statements. The following steps were applied: initial analysis was conducted by reading through the first transcript and developing initial codes and definitions, codes were defined for easier identification, classification and clarification for subsequent transcripts, where necessary new codes were formulated and merged to develop final codes, and themes and sub-themes were developed from the codes and a total of five themes and two sub-themes emerging from the data, which is captured in the thematic index (Table 3):

**TABLE 3:** Thematic index table.

Number	Themes	Sub-themes
1.	Restricted healthcare services during power outages	1.1 Difficulties in registering and accessing records 1.2 Limited access to laboratory results 1.3 Limited access to interventions such as oxygen and surgery 1.4 Limited access to pharmacy
2.	Alternatives available during power outages	2.1 Referral to the hospital 2.2 Backup generators not working
3.	Staff behaviour and attitudes during power outages	3.1 Negative staff attitudes during power outages 3.2 Stressful for staff during power outages
4.	Suggestions to mitigate the impact of power outages	4.1 Previous attempts to engage with staff to improve the situation
5.	Recommended strategies to mitigate the effects of power outages in clinics	5.1 Exempt hospitals and clinics from power outages 5.2 All clinics must have functional generators 5.3 Improve health worker attitudes during power outages: willingness to help 5.4 Clinic committee to improve collaboration and transparency

### Theme 1: Restricted healthcare services during power outages

The first theme that emerged was the disruption in the provision of medical services, as some services were unavailable during power outages.

#### Sub-theme 1.1 Difficulties in registering and accessing records

It was observed that power outage causes delays in essential diagnostic, treatment and administrative processes. Participants complained about the initial registration process, which is affected upon arrival at the clinic during power outages. A 53-year-old woman explained:

‘So, when there’s no electricity, they are unable to register us because the system is not working. So that causes us to stay here for a long time. You woke up so early to get here and you must wait here to get registered, or you must wait until they switch on the generator, the system becomes so slow.’ (Phedisong 4 CHC, participant 1)

This statement was supported by a 30-year-old man who said:

‘When you try to open a file and the computer is off, it’s problematic. It’s problematic because it takes time to load.’ (Soshanguve 3 CHC, participant 2)

The affected electronic systems not only cause interrupted administrative functions, such as registration, as illustrated above, but also affect diagnostic services.

#### Sub-theme 1.2 Limited access to laboratory results

For example, some patients were unable to access their blood results because of power outages. A middle-aged man described his experience saying:

‘Load shedding becomes worse because everything jams. Everything jams like right now we took our cards to the front desk but none of these cards have been called. These cards have codes; to access our blood results, they must check through the computer or through the phone until they get our results. So, without electricity, there is no way they can check, you see?’ (Soshanguve 3 CHC, participant 1)

#### Sub-theme 1.3 Limited access to interventions such as oxygen and surgery

It was further identified that power outages interfere with the functioning of medical equipment responsible for treatment. For example, oxygen therapy was listed as one of the medical services that were most affected, according to participants. A female participant provided an account of how she was not assisted during an emergency, which required oxygen therapy. She explained:

‘Yeah. In short, life stops. But you can’t do anything about it, you can’t move because if you move there is something hindering you here. You try oxygen but if the oxygen is tripping there, it won’t work. Humidifier won’t work. Basically, nothing is going to work, so it’s killing us. Load shedding is killing life.’ (Kgabo CHC, participant 3)

She also talked about how medical operations cannot be performed, saying:

‘When you get to the hospital and there’s a scheduled operation, you can’t even operate because there’s no electricity.’ (Kgabo CHC, participant 3).

#### Sub-theme 1.4 Limited access to pharmacy

Interestingly, one participant elaborated on how the dispensation of medicine is affected, as he indicated:

‘Even now because there’s no electricity it means they’re going to turn us away from this pharmacy because they haven’t even opened. They’re going to turn us away and tell us that we should come at a different day for medication.’ (Shoshanguve 3 CHC, participant 1)

Taking into account the previously mentioned issues, it can be assumed that power outage hinders healthcare facilities’ ability to operate efficiently, leading to delays and disruptions in nearly every aspect of administration, diagnostic and therapeutic services.

### Theme 2: Alternatives available during power outages

The second theme tried to accomplish the second purpose by identifying the various solutions available to CHC patients in the case of a power outage.

#### Sub-theme 2.1 Referral to the hospital

The generator, which is supposed to start when the power goes out, and, in one instance, relocating patients to different medical facilities for further care, were the two main options that the participants presented throughout the interviews. For example, one participant who came during an emergency that required supplemental oxygen treatment was transferred to another healthcare facility because she could not receive any assistance, she explained:

‘I once came here for oxygen and there was no electricity. So, the pump didn’t work, nothing worked; we tried everything at home, so they had to bring me here at the CHC during power outage. And there was nothing they could do. They had to transfer me to the hospital, immediately.’ (Kgabo CHC, participant 3)

Although the generator was one of the choices, running out of diesel for the generator was a typical occurrence, as mentioned by one participant:

‘Yes, there is a generator but even if the generator is their Mam, sometimes there is no petrol.’ (Phedisong 4 CHC, particiapnt 1).

A similar response was given by a 42-year-old female participant who said:

‘Sometimes even the backup generator doesn’t have diesel.’ (Kgabo CHC, partcipant 3)

One of the participants reasoned that:

‘… it may be because of someone not checking if there was enough diesel and poor preparation, that’s what used to happen, that means since there was no power outage, no one was checking the machine if it had diesel or what was happening.’ (Shoshanguve CHC, participant 6)

#### Sub-theme 2.2 Backup generators not working

It was mentioned that this has a negative effect on services because there is no other alternative, and when that does not work, the systems stop. A middle-aged man explained it this way:

‘They didn’t put in measures that when there is no electricity, they should have a Plan B. So, there’s no Plan B, it’s only Plan 1.’ (Phedisong CHC, participant 3)

### Theme 3: Staff behaviour and attitudes during power outages

This theme emerged from recurring statements made by participants based on the attitudes and behaviours of staff during power outages.

#### Sub-theme 3.1 Negative staff attitudes during power outages

There was a general feeling of negative attitudes from staff members during load-shedding as participants perceived them as rude, unwilling to help and disinterested. One participant gave her experience and said:

‘And also when it’s power outage they become discourteous, they change. They don’t address us kindly.’ (Shoshanguve 3 CHC, partcipant 2)

Another participant echoed these words and said:

‘Yeah, these sisters are discourteous’ (Shoshanguve 3 CHC, participant 5)

One of the male participants gave an in-depth explanation of how the nurses can sometimes be dismissive and look uninterested and as if they do not care, he said:

‘So that’s what make people have enemies that are unnecessary. Because someone will talk to you disrespectfully, in any way that they want and you just have to keep quiet. And the main thing is that they don’t know how it affected you and when you get out of the gate, they don’t know what you’re going to do or how you’re going to react. People end up getting hurt because of the way they talk, and they don’t think about how they address people and that what they do, can hurt the next person. They don’t think that the next person also has a mind of their own, you, see?’ (Shoshanguve 3 CHC, participant 6)

Negative attitudes from staff made patients view them as uncaring and uninterested, and some participants indicated:

‘They don’t care about people. They don’t care about anyone.’ (Shoshanguve 3 CHC, participant 6)

Another participant added:

‘They don’t want to work, they are lazy to work!’ (Shoshanguve 3 CHC, participant 5)

#### Sub-theme 3.2 Stressful for staff during power outages

However, some of the other participants understood the stressful situation endured by staff during a power outage, as a participant pointed out:

‘Our sisters and our nurses are struggling really, it’s not like they are not working. They are also under pressure.’ (Kgabo CHC, participant 4)

Someone also highlighted the issue of being short-staffed as she explained:

‘So sometimes there are shortage of sisters at the clinic, they try their best. I really appreciate whatever they do, I really appreciate it.’ (Kgabo CHC, participant 1)

Thus, some of the participants understood the difficulties faced by nursing staff during a power outage, while others felt that the staff was not accommodating, discourteous and unconcerned.

### Theme 4: Suggestions to mitigate the impact of power outages

This theme explored the suggestions made by participants to mitigate the effects of power outages. The first sub-theme was aimed at reviewing whether there were previous attempts made to engage with staff about overall improvements. Moreover, the second sub-theme was specific to potential strategies that can be taken to alleviate the effects of load-shedding.

#### Sub-theme 4.1 Previous attempts to engage with staff to improve the situation

Throughout the progression of the interview, many issues faced by patients came up as they felt they now had a platform to address some of their concerns. For example, a female participant explained:

‘We’ve had a lot of burning issues that we have kept to ourselves and didn’t have anyone to tell. That’s why so many of us are here in this room because we want to talk about all the problems and we’re not just basing this only on issues of electricity, we are also addressing everything.’ (Phedisong 4 CHC, participant 1)

When probed further about whether they had an official platform where they could voice out their concerns, it was revealed that there are currently no available means through which their complaints can be heard. It was indicated that historically, there were suggestion boxes that were available but not anymore. The participants explained that the suggestion boxes were not working, their complaints were not heard, and there were no changes that were implemented. Some of the participants’ responses:

‘To be honest it seems like it’s all the same even with that suggestion box. May be because the people that handle the suggestion box are the same people that work in the clinic, so it needs someone who’s neutral, it was going to be much better. Because honestly, it’s all the same even though there was a suggestion box, anything that we suggest doesn’t go anywhere because we are complaining to the same people. So, there were suggestion boxes, and we used to support them but, in the end, we saw that it‘s all the same. Because we see the same things and it even gets worse. So, it means the suggestion box wasn’t working. That’s why they are no longer around.’ (Phedisong 4 CHC, participant XXX)

‘It wasn’t working for us because we didn’t know who was handling them, how, what time and where.’ (Phedisong 4 CHC, participant 1)

Therefore, the participants felt that the platforms for addressing issues were biased and ultimately useless. Participants were then asked how they think about not having their issues addressed. A male participant complained:

‘There’s nothing you can feel! Because there’s no answer. What can you feel?’ (Shoshaguve 3 CHC, participant 1)

The current reality was that the participants felt as if no one could address their concerns, even those about their discontentment with how services are provided during load-shedding, as it was asked:

‘Who do we raise our concerns to? Who are we going to raise our concerns to? Who is going to listen to us?’ (Phedisong 4 CHC, participant 2)

### Theme 5 Recommended strategies to mitigate the effects of power outages in clinics

This theme explores the strategies recommended by participants on various ways to mitigate the effects of a power outage. There were four main recommendations provided by participants.

#### Sub-theme 5.1 Exempt hospitals and clinics from power outages

The first is targeted at discontinuing power outages in key facilities. The suggestion was made as follows:

‘But I would suggest that in places such as hospitals and clinics our government should make sure that there’s no power outage. Because it’s literally peoples’ lives. They can have their power outage in our homes, that’s fine but not here at the clinic nor in the hospitals. How can they implement power outage in places where they know that people are dying? What are they going to do when there’s someone that needs to be operated right now? I really don’t know how our government can help us.’ (Kgabo CHC, participant 1)

Emphasis on the importance of ensuring that clinics continue to provide services was used as justification to advocate for the discontinuation of the power outage.

#### Sub-theme 5.2 All clinics must have functional generators

The second recommendation suggested producing alternative energy supplies. This was recommended by a participant who believed the current power supply is overburdened; she suggested:

‘I think they need to build more power stations’ (Kgabo CHC, participant 4)

Closely related to this idea was the provision of generators for all clinics, even smaller clinics within the township, to alleviate the burden of larger clinics dealing with a greater population than they can handle. A male participant suggested:

‘Another solution could be if all these clinics can get generators. The focus shouldn’t only be on the clinics. Because this whole problem affects all the clinics in the township. For example, I think few clinics don’t have any generators.’ (Phedisong 4 CHC, participant 3)

Thus, the installation of generators in other clinics will mitigate the issue of load-shedding. It was, however, suggested by the same participant that clinics should always ensure that there is enough diesel for the effective functioning of the generator, as this was the current problem faced in all the clinics:

‘To have a generator is fine, but a working generator full of diesel is the best.’ (Phedisong 4 CHC, participant 2)

#### Sub-theme 5.3 Improve health worker attitudes during power outages: willingness to help

The third suggestion is directed towards addressing staff attitudes by fostering better communication between patients and staff during load-shedding. It was a general feeling that staff did not treat patients well during power outages by being discourteous and not explaining some of the delays experienced. It was said:

‘We don’t want a situation where they say to us, “It’s load shedding what can we do?” Besides load shedding what do you think can happen? Yes, we can see that it is load shedding but if there is a particular type of movement that shows that they are willing to help.’ (Soshanguve 3 CHC, participant 1)

#### Sub-theme 5.4 Clinic committee to improve collaboration and transparency

The participants want to see efforts to try other things on behalf of the staff. Finally, the last suggestion was aimed at implementing an engagement forum where patients can take their complaints. It was proposed:

‘Maybe if they can be sort of like a committee that engages management and patients. That can sit them both down and show each other some of the problems that are faced by patients when they come here. But there must be transparency from the management, there shouldn’t hide anything. I think that’s the way forward that can work for patients.’ (Phedisong 4 CHC, participant 3)

This was said to be a platform where patients can voice out their opinions freely, without the fear of being victimised, to ensure that the issues brought forward are adequately dealt with.

## Discussion

An overview of the study’s findings reveals that participants were generally dissatisfied with the healthcare services provided by Pretoria primary healthcare facilities during power outages. The participants’ emphasis on restricted healthcare services during power outages is in line with a Cape Town report that said South Africa’s ongoing electricity shortages have had a significant negative influence on the country’s healthcare system, resulting in power outages that interfere with vital hospital operations. In addition to critical services like lighting, cooling, ventilation and communications technology, these outages have compromised laboratory and diagnostic equipment, vaccine refrigeration, emergency surgeries and more.^8^

There were limited alternatives available during power outages. This research found two alternative approaches for dealing with power outages: using generators and referring patients to other clinics or hospitals. Although it was not always easy to have a working generator and refer a patient because of a lack of diesel or technical faults with the equipment, as well as the difficulties that patient referral can cause within Pretoria, as explained in Mothupi’s article,^9^ healthcare staff had no choice but to rely on those two backups when in the dark. In contrast to what has been carried out in the Tshwane area or Pretoria, the literature gives alternative approaches to coping with power outages, even though not all of these are applicable in South Africa. Greene suggested seven life-saving solutions for such situations: battery banks, generators, solar power, manual crank generators, wind turbines, hydroelectric power, thermo-electric generators and integrated multiple power solutions.^10^

During power outages, participants perceived the staff workers’ behaviour and attitude were hostile, unprofessional and unfriendly. Some of them handled patient problems in a discourteous manner, which caused tremendous stress to participants (or patients). Other participants attempted to explain how the staff’s attitude and behaviour are related to the pressures of their jobs since they are short-staffed. Such a situation led to overwork and frustration.

To deal with power outages, participants in Pretoria suggested using solar panels and generators. However, as previously said, the usage of generators is hindered by the shortage of diesel and poor planning, particularly the absence of designated staff for diesel restocking and generator maintenance, as suggested by a Malawian study.^4^

During the focus groups, participants highlighted a lack of communication channels, such as suggestion boxes, to voice concerns during power outages, leading to a perception of unheard complaints. This mirrors issues discussed at a nurses’ home in Florida, US.^3^ Suggested mitigation strategies included installing generators in every clinic and exempting facilities providing critical services, like hospitals and clinics, from power outages.^3^

## Conclusion

Community members expressed significant dissatisfaction with health services at three Pretoria CHCs during power outages. Key affected services included administrative functions, diagnostics (e.g. blood results) and treatments (e.g. oxygen therapy). Participants reported long waiting times and negative health outcomes as primary consequences. The limited alternative during outages was generator use, with the perception that staff were unwilling to explore other options. Furthermore, staff attitudes and behaviours during these periods exacerbated the negative experiences.
